# Early Endoscopic Discectomy in Preventing Degenerative Spinal Changes in Patients With Lumbar Disc Herniation

**DOI:** 10.7759/cureus.69725

**Published:** 2024-09-19

**Authors:** Volodymyr Toropchyn, Rohan Sarna, Caitlin M Gray, Sanjeev Kumar

**Affiliations:** 1 Department of Anesthesiology, University of Florida, Gainesville, USA; 2 Division of Pain Medicine, Department of Anesthesiology, University of Florida, Gainesville, USA; 3 Department of Anesthesiology, North Florida/South Georgia Veterans Affairs, Gainesville, USA

**Keywords:** conservative treatment, degenerative spinal changes, endoscopic discectomy, lumbar disc herniation, modic changes

## Abstract

In this study, we compare the outcomes of two patients with similar spinal pathologies who chose different treatment options. The first case involves a 38-year-old female with significant lumbar disc herniation and associated degenerative spinal changes, including type I Modic changes. She presented with sciatica and, after conservative treatments failed, underwent an endoscopic discectomy. This intervention led to a marked improvement in her pain and functional status, along with a partial resolution of the Modic changes and a decrease in multifidus atrophy and fatty infiltration, on her follow-up MRI. In the second case, we discuss a 33-year-old patient with a large disc herniation. Despite three years of conservative management, she developed Modic changes and sclerosis in the adjacent vertebral endplates and a worsening of multifidus atrophy and fatty infiltration. This report supports the consideration of early minimally invasive discectomy for young patients who do not benefit from conservative treatment, as a means to prevent the progression of degenerative spinal changes.

## Introduction

Lumbar disc degenerative disease, including lumbar disc herniation (LDH), is one of the most prevalent causes of lower back pain in the USA and globally [1,2].

This condition not only contributes to a significant burden of morbidity due to its incapacitating pain and functional impairment, but it also provides insight into the complex interplay between mechanical disc disruption and subsequent degenerative changes in the spine.

LDH initiates a cascade of pathologic spine changes by altering the distribution of forces across the vertebral column. It leads to degenerative endplate changes (aka Modic changes) characterized by varying degrees of endplate and bone marrow lesions, Schmorl's nodes indicative of vertical disc material herniation into the vertebral body, facet joint arthropathy as a response to increased loading on the posterior spinal elements, and ligamentum flavum thickening due to compensatory mechanisms against segmental instability [2-7].

A strong association between LDH and other degenerative changes of the spine, including Modic changes, Schmorl’s nodes, facet joint arthropathy, and ligamentum flavum thickening, is well documented [3-6,8].

For example, Mok et al. showed an association between disc degeneration, Modic changes, the presence of Schmorl’s nodes, and historical lumbar injury based on MRI data from 2,449 volunteers [8].

In a histologic study of a total of 917 articular surfaces of lumbar facet joints from cadaveric human spines of younger individuals, Li et al. demonstrated a high incidence of facet degeneration with even early stages of disc degeneration [5].

In a study investigating ligamentum flavum thickness across different degrees of spinal degeneration, it was found that the ligamentum thickness was significantly higher in patients with Pfirrmann grades IV to V degeneration than in those with grades I to III [3]. The Pfirrmann grading system, used to evaluate lumbar intervertebral disc degeneration on MRI, classifies disc degeneration from Grade I (normal) to Grade V (severe degeneration). While Grade I represents a healthy disc with normal height and structure, Grade V, the most severe, is characterized by complete collapse of the disc space, severe structural abnormalities, and extensive loss of signal intensity on MRI. Hence, this study shows a direct correlation between the severity of intervertebral disk degenerative changes and the degree of ligamentum flavum hypertrophy.

Here, we present two illustrative case reports. The first case focuses on a 38-year-old female presenting with sciatica due to severe LDH and resulting type I Modic changes on MRI. In addition to symptomatic relief following endoscopic discectomy, MRI changes following the procedure demonstrated the procedure's impact on the underlying degenerative processes. By contrast, the second case follows a 32-year-old female with a similar presentation both symptomatically and on MRI, who opted for conservative treatment. Her follow-up without discectomy demonstrates a perspective on the development of Modic changes and endplate/vertebral body sclerosis in the context of a significant disc herniation without intervention. The similar initial presentation of these two patients invited comparison. While it is beyond the scope of this case report to declare causation, this juxtaposition fosters a discussion about the benefits of minimally invasive surgery like endoscopic decompression on spinal degeneration, as well as optimal timing for surgical intervention.

## Case presentation

Case 1

A 38-year-old female presented to our clinic with a seven-month history of lower back pain. The patient had a normal BMI of 22 kg/m², had no significant medical or psychiatric history, had never smoked, and had maintained an overall active lifestyle. Initially, she complained of lower back pain radiating to her right leg, which was exacerbated by extended periods of standing. She characterized her pain as hot-burning, shooting, and stabbing, associated with tingling, pins, and needles in her leg. She rated her pain as 10/10 at its worst and 7/10 on average. We recommended physical therapy and prescribed nonsteroidal anti-inflammatory drugs (NSAIDs) and gabapentin to manage her symptoms.

Despite adhering to physical therapy and the medications, her symptoms worsened, leading the physical therapist to suggest pausing the treatment.

At her follow-up visit, the patient reported worsening of her condition, with pain now extending from the lower back to the right posterior thigh, posterolateral thigh, and heel, consistent with the L5 dermatomal distribution.

**Figure 1:**
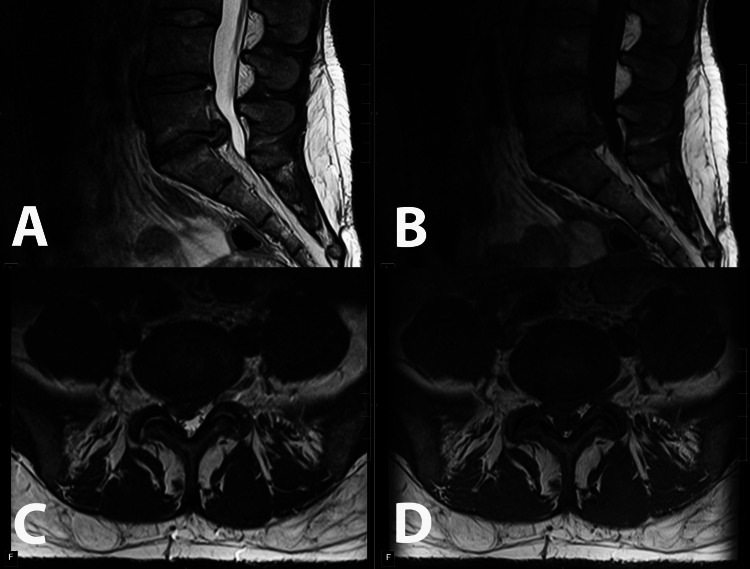
Sagittal T2 (A) and T1 (B) and axial T2 (C) and T1 (D) images demonstrating significant disc herniation at L5-S1 and right lateral recess stenosis. There are associated type I Modic changes of the adjacent L5 and S1 vertebral end plates. Also note the significant atrophy and fatty infiltration of the multifidus muscles bilaterally.

MRI imaging of the lumbar spine revealed significant disc herniation at L5-S1 and right lateral recess stenosis (Figure 1). Following this, she underwent a right transforaminal epidural steroid injection at the L5-S1 level, which provided approximately 25% overall pain relief.

Given the persistent and debilitating nature of her symptoms, the patient elected to proceed with interlaminar endoscopic disc decompression at L5-S1. The surgery aimed to decompress the neural elements, alleviate her right leg pain, and help her return to her previous level of activity.

The procedure was performed with the successful removal of several large disc fragments and decompression of the spinal canal, right L5-S1 lateral recess, the L5 exiting nerve root, and the S1 traversing nerve root. After the procedure, she was discharged home with pain medication for breakthrough pain.

**Figure 2:**
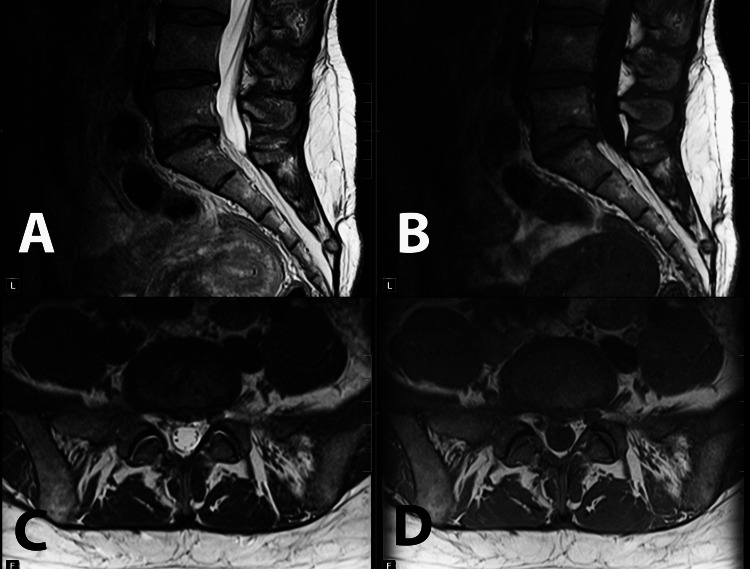
Sagittal T2 (A) and T1 (B) and axial T2 (C) and T1 (D) images showing postsurgical changes with the successful removal of the central disc extrusion and decompression of the L5-S1 space. Visible partial resolution of Modic changes at the adjacent endplates. Please also note the significant decrease in the fatty infiltration of multifidus and a decrease in multifidus atrophy.

At her six-month follow-up visit, she reported a significant reduction in pain, with low back pain at 2/10 at its worst and averaging 1/10, no longer radiating to her right leg. She had ceased taking pain medications. We advised her to continue with the prescribed physical therapy regimen and follow up as needed (Figure 2). The follow-up MRI at six months showed partial resolution of her type 1 Modic changes at the adjacent end plates. The images also showed a significant decrease in fatty infiltration and multifidus atrophy compared to the pre-op MRI.

Case 2

This is a case of a young woman whom we had followed in our clinic for three years.

She initially presented to the clinic at the age of 32 with complaints primarily of acute low back pain. The patient also reported pain and numbness in her right leg. The pain was occurring with standing and prolonged sitting and significantly impacted her daily life. It was associated with numbness in the lateral thigh, extending past the knee to the lateral foot. The pain began five months prior without any preceding injury and progressed from numbness. Initially, she tried meloxicam and had also been undergoing treatment with a chiropractor, massage, and acupuncture, from which she obtained considerable relief overall. At presentation, the patient was physically active, had a BMI of 21, had never smoked, and had no significant past medical or psychiatric history.

**Figure 3:**
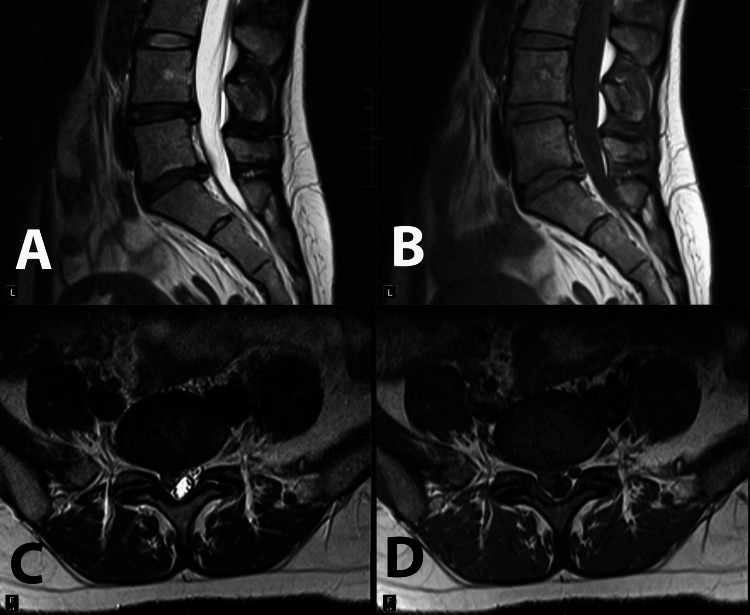
Sagittal T2 (A) and T1 (B) and axial T2 (C) and T1 (D) images of the lumbar spine and L5-S1 intervertebral space, respectively. Significant extrusion at the L5-S1 level, displacing transiting right S1 and S2 nerve roots.

We recommended MRI which demonstrated L4-5 bulge and L5-S1 disc herniation. The more significant extrusion at the L5-S1 level was displacing transiting right S1 and S2 nerve roots (Figure 3).

We recommended the right transforaminal lumbar epidural steroid injection (TFESI) at L5-S1, which led to her pain resolution for a short term, but she continued to have some numbness in her right lower extremity. The patient was not keen to undergo any surgical intervention on her spine since she was aware of all the potential complications of undergoing spine surgery and was scared of the possibility.

The patient returned to the clinic about three years later. Over this period, she continued with conservative treatment including NSAIDs, gabapentin, physical therapy, and home stretching exercises. These interventions provided moderate relief. She noted that her pain has overall gotten worse over time. It still was located at the lower back, traveling to the right posterior lateral thigh, posterior lateral calf to lateral foot, and lateral three toes. More recently, however, she noticed weakness of her right leg and numbness and tingling of her calf and foot. 

**Figure 4:**
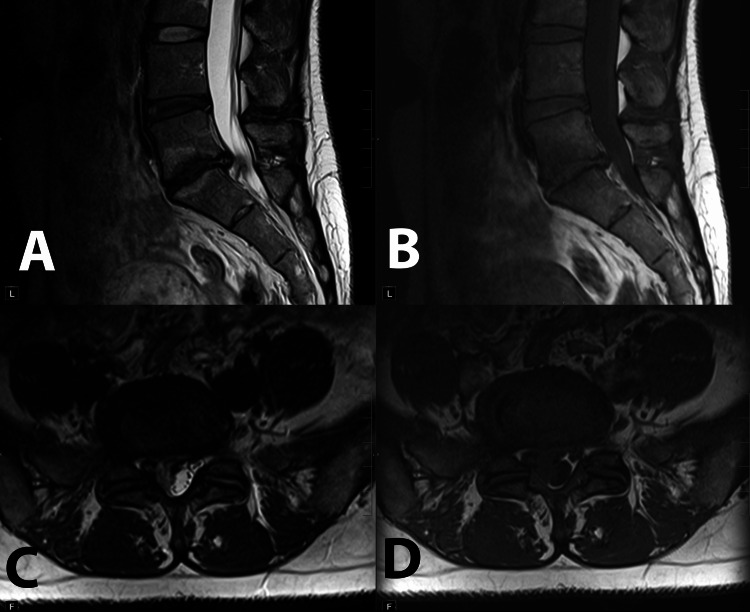
Sagittal T2 (A) and T1 (B) and axial T2 (C) and T1 (D) images of the lumbar spine and L5-S1 intervertebral space, respectively. Progression of the right paracentral L5-S1 disc extrusion with severe narrowing of the right subarticular zone. Type I Modic changes are now clearly identifiable in the adjacent L5 and S1 endplates. Please note the worsening of fatty infiltration and multifidus atrophy.

The repeat MRI showed progression of degenerative spine changes with significant disc extrusion at L5-S1 (Figure 4).

## Discussion

Modic changes represent specific alterations in the vertebral body marrow that are visible on MRI, characterized by distinct MRI signal characteristics. Type 1 Modic changes are indicative of an inflammatory process with hypointense T1 and hyperintense T2 signal changes, suggesting active edema and inflammation. Type 2 Modic changes, characterized by hyperintense T1 and isointense or slightly hyperintense T2 signal changes, reflect fatty degeneration of the marrow. Finally, type 3 Modic changes display both hypointense T1 and T2 signals, indicative of bone sclerosis [9,10].

The development of Modic changes is closely associated with degenerative disc disease and results from structural disruption of the disc/endplate and biomechanical instability [9,11]. This disruption leads to structural failures at the disc-endplate interface, eliciting biological responses that can manifest as inflammation, fatty transformations, or sclerotic changes in the endplates [11-13]. Notably, type 1 Modic changes are more strongly associated with disc herniations, suggesting that the inflammatory responses at the endplate-disc interface due to disc disruption are a common pathway in their pathogenesis [10,11,14].

Moving from Modic changes to another critical aspect of degenerative spine conditions, multifidus atrophy, a prevalent condition in patients with lumbar disc herniation, involves the progressive loss of muscle mass primarily in the multifidus muscles, which is integral to stabilizing the spinal column [15]. The multifidus muscles get innervation from the medial branch of the dorsal ramus of the corresponding spinal nerves [16]. Spinal nerve compression can thereby cause multifidus atrophy, and by the same logic, adequate decompression of the spinal nerves can regain innervation to the multifidus and may reverse multifidus atrophy. The multifidus muscle, when compromised, significantly exacerbates the burden of spinal instability and pain [15,17,18]. Degeneration of these muscles can be assessed on MRI, where decreased muscle size and increased fatty infiltration serve as key indicators [18]. Such atrophy is often linked with chronicity and severity of lower back pain, underscoring the multifidus muscle's role in the functional biomechanics of the spine [15,17].

To quantify the extent of this degeneration, the Goutallier classification system is a well-established method for assessing fatty infiltration within muscles, initially used for the rotator cuff and adapted for spinal musculature evaluation [19]. This classification grades fatty infiltration on a scale from 0 to 4, with higher grades indicating more significant infiltration and muscle degeneration. Grade 0 represents no fatty infiltration, while Grade 4 indicates more than 50% fatty replacement of muscle tissue. This system provides valuable prognostic information, as higher grades of fatty infiltration correlate with poorer clinical outcomes and a higher likelihood of persistent symptoms post-surgery [18].

In the realm of minimally invasive spine surgery, endoscopic discectomy has emerged as a significant advancement [20-22]. Originally conceptualized to minimize disruption during herniated disc material removal, this technique leverages an endoscope to allow for precise visualization and targeted treatment through a minimal incision, preserving surrounding tissues. Over the years, enhancements in optical technology and surgical instruments have significantly increased their safety and efficacy [22-24]. Growing evidence now supports that endoscopic discectomy achieves outcomes comparable to traditional microdiscectomy, with the added advantages of reduced tissue trauma, less postoperative pain, and quicker recovery, aligning well with the objectives of minimally invasive surgery [25-29].

The case presentations in this report highlight the varied clinical decision-making processes in managing severe lumbar disc herniations and associated degenerative changes.

**Figure 5:**
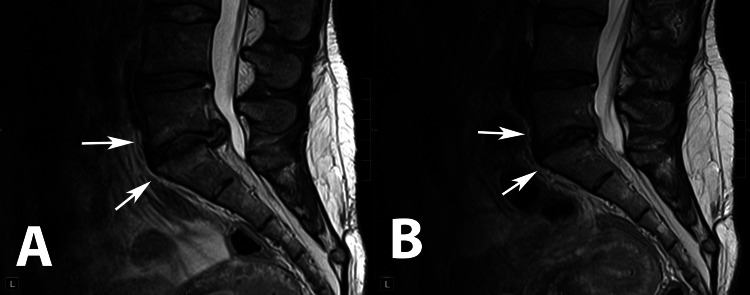
Sagittal T2-weighted MRI images of Patient 1 showing the spine at initial presentation (A) and one year after endoscopic discectomy (B). Evident partial resolution of Modic changes in adjacent L5-S1 vertebral endplates (white arrows).

**Figure 6:**
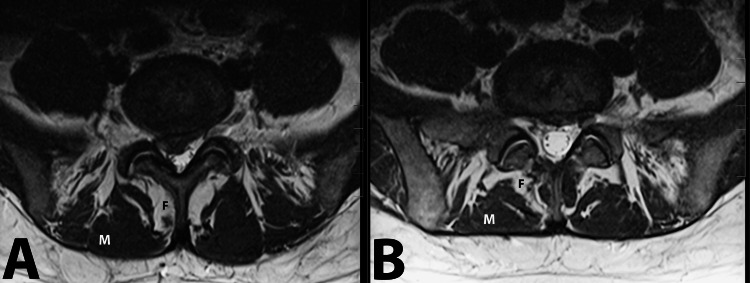
Axial T2-weighted MRI images of Patient 1 showing the spine at initial presentation (A) and one year after endoscopic discectomy (B). Along with the successful decompression of the L5-S1 space, please note the significant decrease in the multifidus muscle fatty infiltration (black letter F) and its atrophy (white letter M).

The first case demonstrates that early intervention with endoscopic discectomy, following the ineffectiveness of conservative treatments, not only alleviates symptoms but also impacts the underlying degenerative processes, as evidenced by the partial reversal of type 1 Modic changes and a decrease in fatty infiltration of multifidus muscle and its atrophy (Figures 5, 6). This suggests that timely and targeted minimally invasive interventions can potentially slow, or even reverse, degenerative changes.

**Figure 7:**
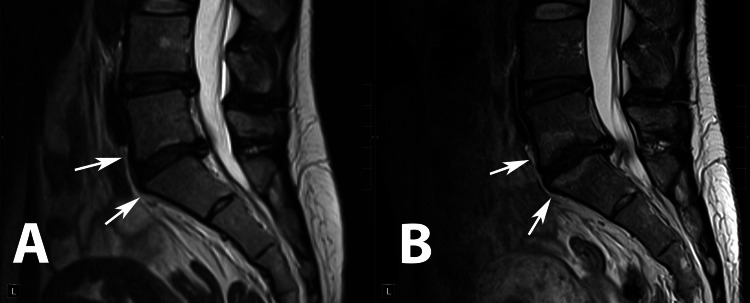
Sagittal T2-weighted MRI images of Patient 2 at initial presentation (A) and three years later. Development of Modic changes in L5, S1 vertebral end plates (white arrows).

**Figure 8:**
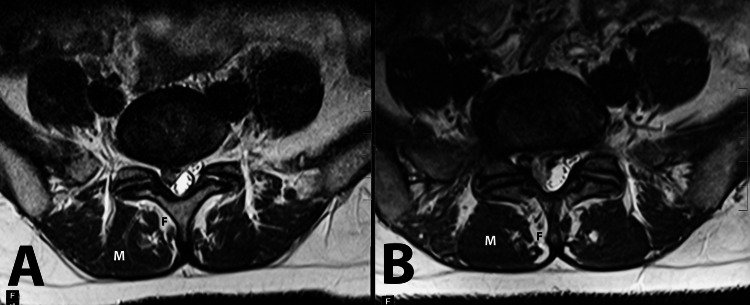
Axial T2-weighted MRI images of Patient 2 at initial presentation (A) and three years later. Note the progression of fatty infiltration (black letter F) and multifidus muscle atrophy (white letter M).

Conversely, the second case underscores the limitations of prolonged conservative management, which, despite initially mitigating symptoms, did not prevent the progression of structural changes such as Modic changes and end-plate sclerosis. Moreover, there was an observable worsening of multifidus muscle atrophy (Figures 7, 8). This case shows that delaying the surgical intervention in the presence of significant disk herniation may lead to the progression of degenerative spine changes.

It is worth noting that both patients had very similar body habitus with BMIs of 22 and 21, respectively. Neither of them ever smoked, and they maintained an active lifestyle, as much as their condition would allow.

One limitation of this report that we acknowledge is the variation in follow-up timelines. While the six-month follow-up MRI for the first patient was available, three-year follow-up imaging would provide a more direct comparison of the two patients' spinal health over time. That said, although the different follow-up timelines may influence the differential progression of degeneration observed on MRI, the reversal of Modic changes and improved multifidus atrophy at the six-month mark are encouraging to the overall trajectory of the patient's spinal health.

## Conclusions

While there are many limitations to this case comparison, these two cases collectively advocate for the consideration of early discectomy in young patients who do not respond adequately to conservative management. Such an approach could potentially forestall the progression of degenerative spinal changes, including Modic changes, and associated multifidus atrophy, thereby preserving spinal architecture and function. At the same time, further research is essential to establish the role of minimally invasive spine surgery in preventing further degenerative changes of the spine.
